# Identification and Characterization of Stimulator of Interferon Genes As a Robust Adjuvant Target for Early Life Immunization

**DOI:** 10.3389/fimmu.2017.01772

**Published:** 2017-12-12

**Authors:** Francesco Borriello, Carlo Pietrasanta, Jacqueline C. Y. Lai, Lois M. Walsh, Pankaj Sharma, David N. O’Driscoll, Juan Ramirez, Spencer Brightman, Lorenza Pugni, Fabio Mosca, David J. Burkhart, David J. Dowling, Ofer Levy

**Affiliations:** ^1^Division of Infectious Diseases, Department of Medicine, Boston Children’s Hospital, Boston, MA, United States; ^2^Harvard Medical School, Boston, MA, United States; ^3^Precision Vaccines Program, Divisions of Infectious Diseases, Boston Children’s Hospital, Boston, MA, United States; ^4^Department of Translational Medical Sciences, Center for Basic and Clinical Immunology Research (CISI), University of Naples Federico II, Napoli, Italy; ^5^WAO Center of Excellence, Naples, Italy; ^6^Neonatal Intensive Care Unit, Department of Clinical Sciences and Community Health, Fondazione IRCCS Ca’ Granda Ospedale Maggiore Policlinico, Università degli Studi di Milano, Milan, Italy; ^7^Department of Physiology, Institute of Neuroscience and Physiology, University of Gothenburg, Gothenburg, Sweden; ^8^Biomedical & Pharmaceutical Science Skaggs School of Pharmacy, University of Montana, Missoula, MT, United States

**Keywords:** vaccines, adjuvants, newborn, antigen-presenting cells, germinal centers, T follicular helper cells, antibodies, stimulator of interferon genes

## Abstract

Immunization is key to preventing infectious diseases, a leading cause of death early in life. However, due to age-specific immunity, vaccines often demonstrate reduced efficacy in newborns and young infants as compared to adults. Here, we combined *in vitro* and *in vivo* approaches to identify adjuvant candidates for early life immunization. We employed newborn and adult bone marrow-derived dendritic cells (BMDCs) to perform a screening of pattern recognition receptor agonists and found that the stimulator of interferon genes ligand 2′3′-cGAMP (hereafter cGAMP) induces a comparable expression of surface maturation markers in newborn and adult BMDCs. Then, we utilized the trivalent recombinant hemagglutinin (rHA) influenza vaccine, Flublok, as a model antigen to investigate the role of cGAMP in adult and early life immunization. cGAMP adjuvantation alone could increase rHA-specific antibody titers in adult but not newborn mice. Remarkably, as compared to alum or cGAMP alone, immunization with cGAMP formulated with alum (Alhydrogel) enhanced newborn rHA-specific IgG2a/c titers ~400-fold, an antibody subclass associated with the development of IFNγ-driven type 1 immunity *in vivo* and endowed with higher effector functions, by 42 days of life. Highlighting the amenability for successful vaccine formulation and delivery, we next confirmed that cGAMP adsorbs onto alum *in vitro*. Accordingly, immunization early in life with (cGAMP+alum) promoted IFNγ production by CD4^+^ T cells and increased the proportions and absolute numbers of CD4^+^ CXCR5^+^ PD-1^+^ T follicular helper and germinal center (GC) GL-7^+^ CD138^+^ B cells, suggesting an enhancement of the GC reaction. Adjuvantation effects were apparently specific for IgG2a/c isotype switching without effect on antibody affinity maturation, as there was no effect on rHA-specific IgG avidity. Overall, our studies suggest that cGAMP when formulated with alum may represent an effective adjuvantation system to foster humoral and cellular aspects of type 1 immunity for early life immunization.

## Introduction

Infectious diseases represent a major cause of morbidity and mortality in neonates and young infants (1, 2). For example, each year in the US ~20,000 children <5 years old are hospitalized due to influenza complications and flu-related death may occur, especially among those with underlying chronic illness (3). Immunization strategies are fundamental to prevent infectious diseases. However, due to age-specific immunity, vaccines often demonstrate reduced efficacy in newborns and young infants compared to adults (4, 5). Newborn innate immune cells exhibit distinct activation profiles in response to pattern recognition receptor (PRR) agonists (6, 7), and only certain PRR agonists (e.g., TLR7/8 agonists) (8–14) or their combinations (15, 16) are able to induce an adult-like response. The newborn adaptive immune compartment presents distinct features that may also limit vaccine efficacy. Neonatal B cells can produce immunoregulatory cytokines (e.g., IL-10) (17–20), and the magnitude and persistence of the antibody response are reduced (21). Several mechanisms may contribute to distinct immunity in early life, including distinct activity of B and plasma cells (22, 23), the presence of maternal antibodies, impaired CD4^+^ CXCR5^+^ PD-1^+^ T follicular helper (Tfh) cell differentiation and lymph node germinal center (GC) reaction (24, 25) that may adequately support the antigen-specific B cell response. Moreover, neonatal CD4^+^ T cells produce lower amounts of IFNγ and are skewed toward Th2, Th17, and Treg polarization (6, 7). Of note, adjuvants exhibit age-specific patterns of Th-polarization (16) such that adjuvantation systems that boost adult immune responses do not necessarily lead to enhanced vaccine efficacy in newborns or young infants (26). Therefore, identification of vaccine adjuvants capable of activating neonatal and infant immune responses may inform development of adjuvanted vaccine formulations that enhance early life immunization (8, 9).

Dendritic cells (DCs) play a pivotal role in activating T cells and instructing the adaptive immune response. They express a high diversity of PRRs, whose activation leads to DC migration to lymph nodes and enhancement of immune-stimulatory functions (27). Recently, a systems vaccinology analysis of young infants vaccinated with trivalent inactivated influenza vaccine with or without the oil-in-water adjuvant MF59 demonstrated that innate immune gene signatures (e.g., antiviral and DC genes) 1 day post-immunization correlated with vaccine efficacy, highlighting the importance of robust innate immune activation in early life immunization (28). Agonists of the intracellular receptors TLR7/8, that recognize viral single-stranded RNAs, potently activate Th1-polarizing responses, including expression of interferons (IFNs), production of IL-12p70 and upregulation of co-stimulatory molecules in newborn DCs *in vitro* and enhance vaccine efficacy in newborn non-human primates *in vivo* (8–14). Moreover, adjuvantation with the TLR9 agonist CpG increases CG Tfh and B cell responses in newborn mice (25). Among intracellular PRRs, the stimulator of interferon genes (STING) is an amenable target for adjuvant discovery and development (29, 30). It binds cyclic dinucleotides (CDNs) derived from bacteria (i.e., c-di-AMP, c-di-GMP, and 3′3′-cGAMP) or synthesized in mammalian cells by cGAMP synthase in response to double-stranded DNA in the cytoplasm (i.e., 2′3′-cGAMP). Upon activation, STING induces the TBK-1-mediated phosphorylation of IRF3, which in turn modulates the expression of type I IFNs, IFN-stimulated genes, and also promotes DC maturation and type 1 (i.e., IFNγ-driven) immunity (31). Accordingly, STING agonists have demonstrated promising adjuvanticity in adult experimental models of parenteral and mucosal immunization as well as cancer immunotherapy (32–49). However, to our knowledge, STING has not yet been investigated as an adjuvant target for early life immunization.

Here, we took an unbiased approach to identify PRR-based agonists for early life immunization. We employed adult and neonatal bone marrow-derived DCs (BMDCs) to screen the activity of a comprehensive panel of PRR agonists and adjuvants, and found that the STING ligand 2′3′-cGAMP is a potent activator of newborn BMDCs. Strikingly, we found that 2′3′-cGAMP formulated with alum induces antibody isotype switching toward IgG2a/c, a subclass endowed with higher effector functions, appears to enhance the GC reaction and also promotes Th1 polarization in immunized newborn mice. Altogether, our study supports the use of STING ligands and their formulations for enhancement of early life immunization.

## Materials and Methods

### Ethics Statements

All experiments involving animals were approved by the Animal Care and Use Committee of Boston Children’s Hospital and Harvard Medical School (protocol numbers 15-11-3011 and 16-02-3130).

### Animals

C57BL/6 and BALB/c mice were obtained from Taconic Biosciences or Charles River Laboratories and housed in specific pathogen-free conditions in the animal research facilities at Boston Children’s Hospital. For breeding purposes, mice were housed in couples, and cages checked daily to assess pregnancy status of dams and/or the presence of pups. When a new litter was discovered, that day was recorded as day of life (DOL) 0. Both male and female pups were used for experiments.

### Generation of Neonatal and Adult Murine Bone Marrow-Derived Dendritic Cells (BMDCs)

BMDCs were generated from newborn (5–7 days old) and adult (6–12 weeks old) C57BL/6 mice with an adaptation of previously described methods (50, 51). Briefly, mice were sacrificed and legs removed; bones were surgically cleaned from surrounding tissue, extremities of tibiae and femurs were trimmed with sterile scissors and bone marrow flushed through a 70-µm nylon mesh strainer (Corning Life Sciences). Cell number and viability was determined by trypan blue exclusion. Whole bone marrow cells were plated into non-tissue culture-treated 100 mm Petri dishes (Corning Life Sciences) at a density of 0.3 × 10^6^ cells/ml in 10 ml total volume/plate of complete culture medium (RPMI 1640 plus 10% heat-inactivated fetal bovine serum [FBS, GE Healthcare HyClone], 50 µM 2-mercaptoethanol, 2 mM l-glutamine, 100 U/ml penicillin/streptomycin [Gibco ThermoFisher Scientific]) supplemented with 20 ng/ml of recombinant murine GM-CSF (rmGM-CSF, R&D systems). Plates were incubated in humidified atmosphere at 37°C, 5% CO_2_ for 6 days, with one supplement of 10 ml of complete culture medium and rmGM-CSF on day 3. On day 6, non-adherent and loosely adherent cells were harvested by washing the plate gently with culture medium. Adherent cells were discarded. For flow cytometry analysis, BMDCs were stained (20 min at 4°C) in PBS + FBS 2% + EDTA 2 mM, fixed with formaldehyde 4% [10 min at room temperature (RT)] and acquired on a BD LSRFortessa flow cytometer (BD Biosciences) or a Sony spectral analyzer SP6800 (Sony Biotechnology) and data were analyzed using FlowJo v.10 software (Tree Star). For a complete list of antibodies and fluorochromes used in the study, see Table S1 in Supplementary Material.

### PRRs Agonists, Adjuvants, and BMDC *In Vitro* Stimulation

Rough (*Salmonella Minnesota*, R595) and smooth (*Escherichia coli*, O55:B5) lipopolysaccharide (LPS) were purchased from List Biological Laboratories. Aluminum hydroxide (Alhydrogel) and Aluminum phosphate (Adju-phos) were purchased from Brenntag Biosector. All remaining PRR agonists and adjuvants, as indicated in Table S2 in Supplementary Material, were purchased from Invivogen. All PRR agonists employed in the studies were chosen based on and verified endotoxin free as indicated by the manufacturers. For stimulation experiments, immature BMDCs generated from newborn and adult mice were plated in round bottom 96-wells non-tissue culture-treated plates at the density of 10^5^ cells/well in 200 µl of fresh complete culture medium with rmGM-CSF as described above, with the appropriate stimuli at the concentrations indicated in Table S2 in Supplementary Material. Cells were incubated at 37°C for 20–24 h, then supernatant harvested and TNF, IL-6, IL-1β, and IL-12p70 concentrations were measured by ELISA (R&D Systems). IFNβ was measured with a bioluminescent ELISA kit (LumiKine, Invivogen). Alternatively, BMDCs were stained and analyzed by flow cytometry as indicated above. For experiments involving blocking antibodies, BMDCs were pre-incubated for 20 min at 37°C with anti-mouse IFNAR1 (clone MAR1-5A3, 10 µg/ml, Biolegend) or anti-mouse TNF (clone MP6-XT22, 10 µg/ml, Biolegend) antibodies or an isotype control before stimulation.

### Antigens, Immunization, and Antibody Quantification

Both neonate and adult mice were immunized intramuscularly (i.m.) in the right posterior thigh with 50 µl of the 2016–2017 formulation of the FluBlok vaccine (Protein Sciences Corp.) containing 0.33 µg of each of the following recombinant influenza virus hemagglutinins (rHA): A/Michigan/45/2015 (H1N1), A/Hong Kong/4801/2014 (H3N2), and B/Brisbane/60/2008. Mice were immunized with a single dose at DOL 7 or a prime-boost schedule (two injections 1 week apart, for newborn mice at DOL 7 and 14). As indicated for specific experimental groups, the vaccine was formulated with Aluminum hydroxide (100 µg, hereafter “alum”) with or without 2′3′-cGAMP (10 µg). Serum was collected at the indicated intervals for antibody detection. rHA-specific IgG, IgG1, IgG2c (for C57BL/6 mice), and IgG2a (for BALB/c mice) antibodies were quantified by ELISA. High binding flat bottom 96-well plates (Corning Life Sciences) were coated with 1 µg/ml rHA in carbonate buffer pH 9.6, incubated overnight at 4°C and blocked with PBS + BSA 1% (Sigma-Aldrich) for 1 h at RT. Then, sera from vaccinated mice were added with an initial dilution of 1:100 and 1:4 serial dilutions in PBS + BSA 1% and incubated for 2 h at RT. Plates were then washed and incubated for 1 h at RT with HRP-conjugated anti-mouse IgG, IgG1, IgG2c, or IgG2a (Southern Biotech). At the end of the incubation, plates were washed again and developed with tetramethylbenzidine (BD Biosciences) for 5 min, then stopped with 2 N H_2_SO_4_. The optical density was read at 450 nm Versamax microplate reader with SoftMax Pro Version 5 (both from Molecular Devices) and endpoint titers were calculated using as cutoff three times the optical density of the background.

For assessing antibody avidity, plates were incubated 15 min with ammonium thiocyanate 0.5 M before the addition of HRP-conjugated anti-mouse IgG antibodies. Avidity was expressed as the LogEC_50_ ratio of corresponding plates treated with or without ammonium thiocyanate.

### Quantification of 2′3′-cGAMP Adsorption onto Alum

To quantify the extent of 2′3′-cGAMP adsorption to aluminum hydroxide (Alhydrogel) we mixed 100 µg/100 μl of 2′3′-cGAMP with 1000 µg/100 μl of alum (a 1:10 cGAMP:alum mass ratio) plus 300 µl of 0.9% saline. After vortexing for 10 s the sample was placed in a 37°C incubator. Every 15 min, the sample was vortexed for an additional 5 s and placed back into the incubator. Aliquots were taken at t = 0.25, 0.5, 1, 2, 4 and 24 h and centrifuged at 3,000 RPM (rcf = 664 g) to separate the alum from the supernatant. Supernatant was immediately removed and placed into an autosampler vial undiluted for analysis by reverse-phase high-performance liquid chromatography (RP-HPLC) to determine adsorption as a function of time. RP-HPLC samples were run on a Waters 2695 HPLC equipped with a 2996 photodiode array detector at a wavelength of 254 nm. A gradient was performed using a two mobile phase system of 0.1% trifluoroacetic acid in water and 0.1% trifluoroacetic acid in acetonitrile, on an Agilent Zorbax Eclipse Plus C18, 4.6 × 150 mm, 5 µm column at 25°C. The response (peak area) of the samples were compared against a 50 µl 2′3′-cGAMP plus 200 µl 0.9% saline control and a separate 100 µl alum plus 400 µl saline control.

### *In Vitro* Restimulation of rHA-Specific T Cell Responses

Splenocytes from immunized mice were harvested 10 days post-boost (DOL 24) as previously reported (25, 52, 53) and re-stimulated *in vitro* to assess cytokine production by flow cytometry. Spleens were mashed through a 70 µM strainer, washed with PBS, and erythrocytes were lysed with 2 min of incubation in ammonium chloride-based lysis buffer (BD Biosciences). Cells were then counted and plated 2 × 10^6^ per well (round bottom 96-well plate) in 200 µl of complete culture medium with or without rHA 10 µg/ml or rHA 10 µg/ml + anti-mouse CD28 2 µg/ml (BioLegend). Plates were incubated for 18 h at 37°C with the addition of Brefeldin A (BD Biosciences) for the last 6 h. Cells were stained against for surface antigens in (PBS + BSA 0.2% + NaN_3_ 0.05%) for 20 min at 4°C, then fixed with formalin 2% (10 min at RT) and permeabilized with intracellular staining permeabilization wash buffer (BioLegend) for 20 min at 4°C. Finally, cells were stained with conjugated antibodies against IFNγ, IL- 2, IL-4, and IL-17. Data were acquired on a BD LSRFortessa flow cytometer (BD Biosciences) and analyzed using FlowJo v.10 software (Tree Star). For a complete list of antibodies and fluorochromes used in the study, see Table S1 in Supplementary Material.

### Analysis of the GC Reaction

Draining (inguinal) lymph nodes (dLNs) from immunized mice were harvested 10 days post-boost (DOL 24) as previously reported (25, 52, 53). To prepare a single-cell suspension, dLNs were pressed using the plunger end of a syringe. Then, cells were washed and stained with the following antibodies: for GC Tfh cells, anti-CD45, anti-B220, anti-CD3, anti-CD4, anti-programmed death-1 (CD279 or PD-1), anti-CXCR5; for GC B cells, anti-CD45, anti-B220, anti-CD3, anti-GL7, and anti-Syndecan-1 (CD138) (all from BioLegend). GC Tfh cells were defined as viable singlet CD45^+^ B220^−^ CD3^+^ CD4^+^ CXCR5^+^ PD-1^+^ cells. GC B cells were defined as viable singlet CD45^+^ B220^+^ CD3^−^ CD138^−^ GL-7^+^. Cells were acquired on a BD LSRFortessa (BD Biosciences) and data were analyzed using FlowJo v.10 software (Tree Star). Absolute number of cell subsets were determined using CountBright Absolute Counting Beads (ThermoFisher Scientific). For a complete list of antibodies and fluorochromes used in the study, see Table S1 in Supplementary Material.

### IFNγ ELISPOT

Draining lymph nodes from immunized mice were harvested 3 days post-boost (DOL 17). Nitrocellulose 96-microwell plates (Millipore) were coated with 75 µl/well of anti-mouse IFNγ (10 µg/ml in PBS, clone R4-6A2, BD Pharmingen) overnight at 4°C, washed twice with wash buffer (PBS + Tween-20 0.05%) and once with distilled water. Wells were blocked with 200 µl of complete culture medium for 2 h at RT. Single-cell suspensions of dLNs in complete culture medium supplemented with recombinant mouse IL-2 (5 ng/ml, PeproTech) were added to the wells in the presence or absence of 10 µg/ml of Flublok and 2 µg/ml anti-mouse CD28 (Biolegend) and cultured for 18 h. Wells were then washed and incubated with 100 ml of biotinylated anti-mouse IFNγ (5 µg/ml in PBS + FBS 10%, clone XMG1.2, BD Pharmingen) for 2 h at RT, washed again and incubated with 100μl of streptavidin-alkaline phosphatase (1:1000 dilution in PBS + FBS 10%, MabTech) for 1 h prior to color development using BCIP/NBT substrate (Biorad) as per manufacturer’s protocol. Spots on air-dried plates were counted on an ImmunoSpot Analyzer.

### Statistical Analyses and Graphics

Data were analyzed and graphed using Prism for MacIntosh v. 7.0 (GraphPad Software). Tests used for statistical comparisons are indicated in figure legends. *p*-value <0.05 was considered significant.

## Results

### Phenotypic and Functional Characterization of Neonatal BMDCs

Murine BMDCs represent a widely used model to study DC function *in vitro*. Adult BMDCs represent a heterogeneous population composed of CD11c^+^ macrophage-like and DC-like cells with distinct phenotypic and functional profiles (54). However, murine neonatal BMDCs have never been characterized in depth. Therefore, we first sought to define the phenotypic and functional properties of neonatal BMDCs. Although the cell yield from neonatal bone marrow was lower compared to adult ones (Figures S1A,B in Supplementary Material), neonatal immature BMDCs generated from 7-day-old mice grew in culture similarly to adult cells (Figure S1C in Supplementary Material), and once fully differentiated they expressed similar levels of CD11c compared to adult cells but significantly lower levels of MHCII (Figures S1D,E in Supplementary Material). To further characterize phenotypic differences between newborn and adult BMDCs, we assessed by flow cytometry the expression of different macrophage and DC markers. As previously reported for adult BMDCs (54), neonatal BMDCs also comprised CD11c^+^ MHCII-low and CD11c^+^ MHCII-high cells. Of note, the percentage of MHCII-low cells was higher in neonatal BMDCs compared to adult BMDCs. Neonatal MHCII-low BMDCs also expressed higher levels macrophage-associated markers (CD64, CD115, CD11b, F4/80) compared to MHCII-high BMDCs, while this population expressed higher levels of CD117. No significant differences in surface marker expression were found between corresponding neonatal and adult MHCII-high and -low populations, except for neonatal MHCII-low BMDCs that expressed higher levels of F4/80 and neonatal MHCII-high BMDCs that expressed higher levels of CD117 compared to their adult counterparts (Figures S2A,B in Supplementary Material).

To characterize a functional response of newborn BMDCs, we next assessed cytokine production and upregulation of co-stimulatory molecules in response to the TLR4 agonist smooth LPS. While newborn BMDC production of IL-6 and TNF was, respectively, comparable or slightly lower than adult BMDCs, IL-12p70 production, albeit detectable, was markedly reduced compared to adult BMDCs (Figure S3A in Supplementary Material). The latter result might be consistent with a more macrophage-like phenotype of newborn BMDCs. As previously reported, both adult and newborn BMDCs produced IL-1β in response to rough but not smooth LPS (55), with newborn BMDCs producing slightly higher amounts of IL-1β (Figure S3B in Supplementary Material). Finally, newborn BMDCs expressed lower levels of MHCII, CD40, and CD86 in response to smooth LPS (Figures S3C,D in Supplementary Material).

### Identification of STING As a Target for Inducing Neonatal BMDC Maturation

**Figure 1:**
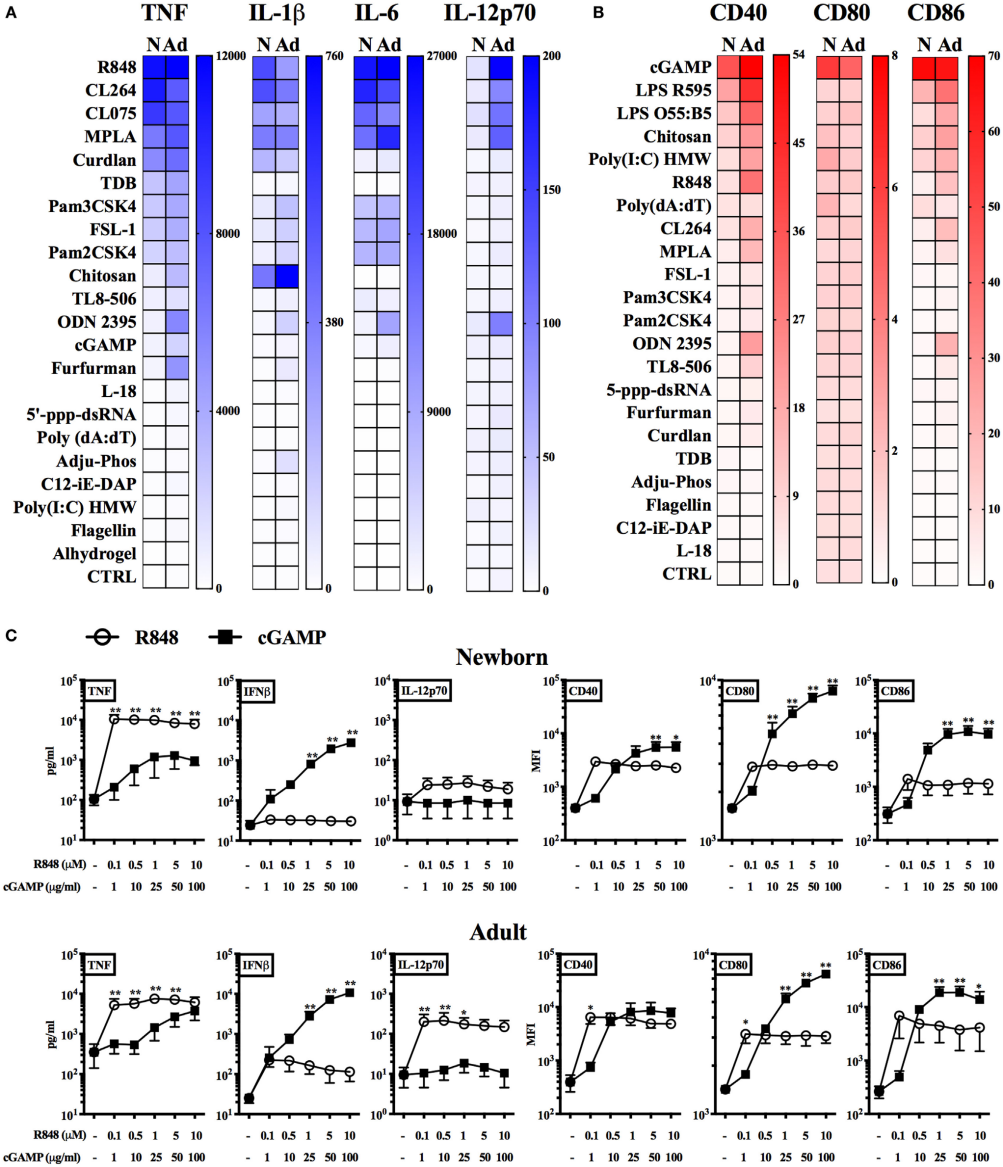
Screening of pattern recognition receptor (PRR) agonists on neonatal and adult BMDCs. **(A–C)** Newborn (N) and adult (Ad) BMDCs were stimulated with the indicated PRR agonists or adjuvants for 20–24 h. Cytokine production **(A,C)** and MFI of surface marker expression **(B,C)** were, respectively, assessed by ELISA and flow cytometry. **(A,B)** Color intensities of the heatmaps are proportional to **(A)** mean cytokine levels (expressed as pg/ml) or **(B)** mean co-stimulatory molecule levels (expressed as fold change of median fluorescence intensity over CTRL) of 5–6 **(A)** or 3 **(B)** independent experiment. **(C)** Results are expressed as mean + SEM of 4–5 (cytokine production) or 3 (surface marker expression) independent experiments. *p < 0.05, **p < 0.01 determined by repeated measures two-way ANOVA with Sidak *post hoc* test.

Having characterized phenotypic and functional features of neonatal and adult BMDCs, we next assessed their response to a panel of PRR agonists and adjuvants (Table S2 in Supplementary Material). As readouts we measured cytokine production (TNF, IL-1β, IL-6, and IL-12p70) and surface expression of maturation markers (CD40, CD80, and CD86). At the most effective, non-toxic (as established in preliminary experiments, data not shown) concentration of each agonist (in bold in Table S2), neonatal BMDCs produced similar amounts of TNF, IL-6, and IL-1β compared to adult BMDCs in response to different TLR7/8 agonists, namely R848 (Resiquimod, imidazoquinoline), CL075 (thiazoloquinolone) or CL264 (9-benzyl-8 hydroxyadenine), but again failed to produce IL-12p70 (Figure 1A). Remarkably, the upregulation of surface maturation marker expression on neonatal BMDCs was much lower than adult BMDCs upon any PRR stimulation, with the exception of the STING agonist 2′3′-cGAMP (hereafter cGAMP) (Figure 1B). To assess in depth the response to STING and TLR7/8 agonists, we stimulated neonatal and adult BMDCs with different concentrations of cGAMP and R848. We confirmed that R848 induced higher production of TNF and IL-12p70 (the latter only in adult BMDCs), while cGAMP was more effective than R848 at upregulating the expression of surface maturation markers (Figure 1C). cGAMP also induced dose-dependent IFNβ production in both newborn and adult BMDCs (Figure 1C). Of note, the response of neonatal and adult BMDCs to cGAMP was comparable (Figure S4 in Supplementary Material). Using neutralizing antibodies against TNF or type I IFN receptor (IFNAR), we demonstrated that the expression of maturation markers by neonatal BMDCs mostly relies on type I IFN signaling (Figure S5 in Supplementary Material).

### cGAMP Formulated with Alum Enhances Anti-rHA IgG2a/c Antibody Titers in an Early Life Immunization Model

**Figure 2:**
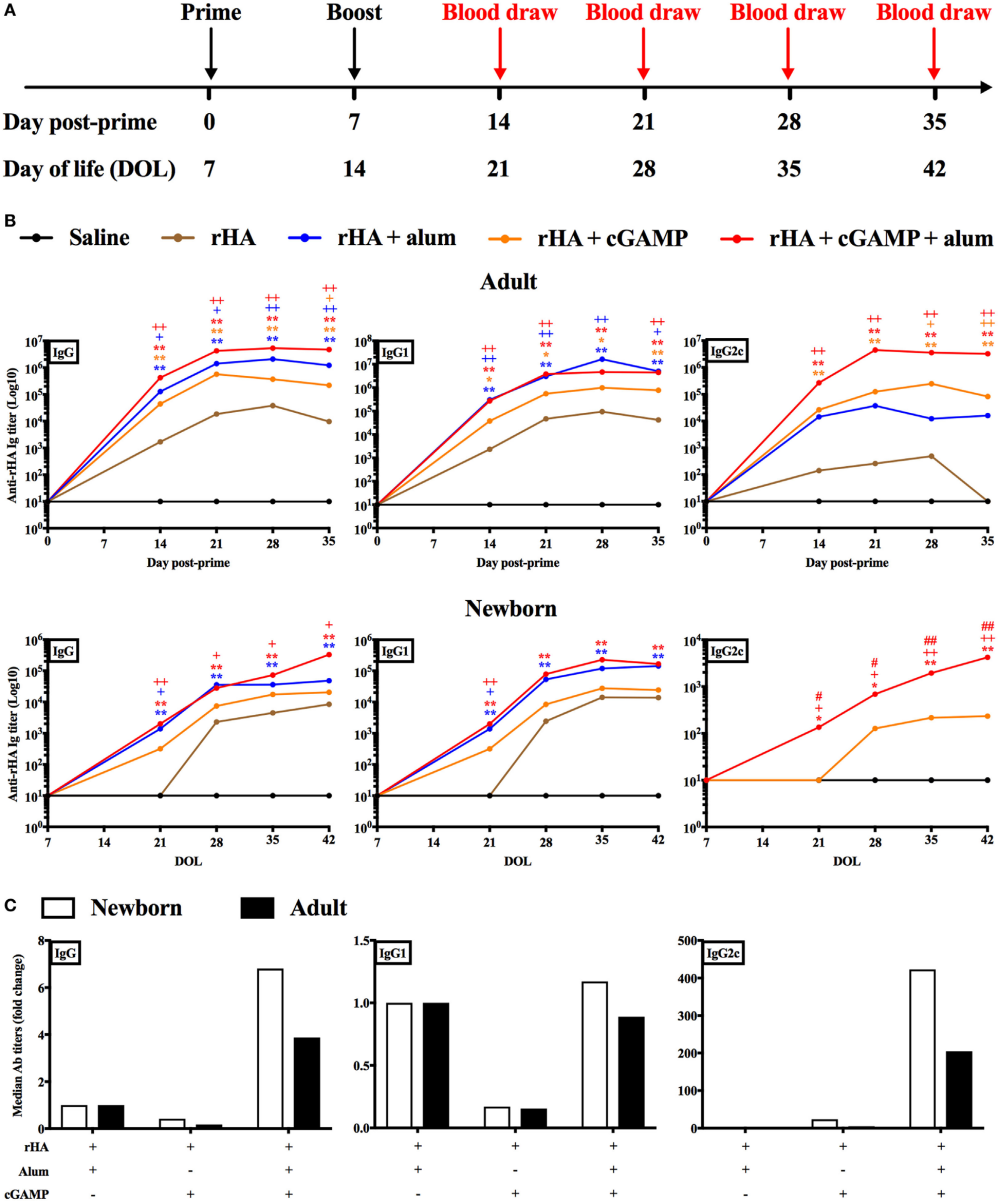
Immunization with recombinant hemagglutinin (rHA) formulated with cGAMP and alum induces distinct antibody profiles in adult and newborn mice. **(A)** Schematic representation of the immunization schedule for adult (day post-priming is indicated) and newborn [day of life (DOL) is indicated] mice. **(B)** Adult (top) and newborn (bottom) mice were immunized i.m. with saline (black line), rHA (brown line), (rHA + alum) (blue line), (rHA + cGAMP) (orange line) or (rHA + cGAMP + alum) (red line), and antibody titers for rHA-specific IgG, IgG1, and IgG2c were determined by ELISA in serum samples collected at the reported timepoints. **(C)** Fold change of median Ab titers over (rHA + alum) group. White bars, newborn mice. Black bars, adult mice. Results are shown as median of 9–10 (adult) or 7–8 (newborn) mice per group. *, ^+^, ^#^*p* < 0.05, **, ^++^, ^##^*p* < 0.01 of groups indicated by the corresponding color, respectively, vs. saline, rHA, and (rHA + alum) groups determined by Kruskal–Wallis with Dunn’s *post hoc* test.

The *in vitro* results obtained so far supported further investigation of cGAMP as adjuvant candidate for early life immunization. Therefore, we proceeded to test this hypothesis *in vivo*. We immunized newborn (7-day old) and adult (8- to 10-week old) C57BL/6 mice using a prime-boost schedule (Figure 2A) and employing trivalent recombinant hemagglutinin (rHA) influenza vaccine Flublok as clinically relevant model antigen that is devoid of adjuvant, alone, or formulated with alum [Alhydrogel, Al(OH)3], cGAMP or (cGAMP + alum) (Figure 2B). Mice were bled 14, 21, 28, and 35 days post-prime (respectively, day of life (DOL) 21, 28, 35, and 42 for newborn mice) to assess the magnitude and kinetic of the antibody response. As expected, both alum and cGAMP increased anti-rHA IgG titers in adult mice. We also investigated the titers of the IgG subclasses IgG1 and IgG2c, respectively associated with type 2 and type 1 (IFNγ-driven) immunity (56, 57). In keeping with previously published data, alum preferentially increased anti-rHA IgG1 titers (median anti-rHA IgG1 titers at Day 35 post-prime: 5.02 × 10^6^ for alum, 0.77 × 10^6^ for cGAMP), while cGAMP was more effective than alum at enhancing anti-rHA IgG2c titers (median anti-rHA IgG2c titers at day 35 post-prime: 0.16 × 10^6^ for alum, 0.82 × 10^6^ for cGAMP). (cGAMP + alum) was as effective as alum at increasing anti-rHA IgG and IgG1 titers [median anti-rHA IgG and IgG1 titers at day 35 post-prime: respectively, 4.77 × 10^6^ and 4.46 × 10^6^ for (cGAMP + alum)], and even more effective than cGAMP alone at enhancing anti-rHA IgG2c titers [median anti-rHA IgG2c titers at day 35 post-prime: 3.27 × 10^6^ for (cGAMP + alum)] (Figure 2B, upper panels and Figure S6 in Supplementary Material). In newborn mice, we unexpectedly found that cGAMP was much less effective at increasing anti-rHA IgG, IgG1, and IgG2c titers [median anti-rHA IgG, IgG1, and IgG2c titers at day 35 post-prime (DOL 42): respectively, 20.57 × 10^3^, 24.51 × 10^3^, and 0.23 × 10^3^ for cGAMP]. Alum enhanced anti-rHA IgG and IgG1 titers, but in marked contrast from adult mice it did not induce anti-rHA IgG2c titers [median anti-rHA IgG, IgG1, and IgG2c titers at day 35 post-prime (DOL 42): respectively, 48.35 × 10^3^, 143.23 × 10^3^, and 0.00 × 10^3^ for alum]. Surprisingly, (cGAMP + alum) adjuvantation matched or exceeded alum at increasing anti-rHA IgG and IgG1 titers [median anti-rHA IgG and IgG1 titers at Day 35 post-prime (DOL 42): respectively, 329.19 × 10^3^ and 167.83 × 10^3^ for (cGAMP + alum)], and, remarkably, also induced relatively high titers of anti-rHA IgG2c as early as 14 days post-prime (DOL 21) [median anti-rHA IgG2c titers at day 14 (DOL 21) and day 35 post-prime (DOL 42): respectively, 0.14 × 10^3^ and 4.23 × 10^3^ for (cGAMP + alum)] (Figure 2B, lower panels and Figure S7 in Supplementary Material). Therefore, the addition of cGAMP to alum markedly enhanced anti-rHA antibody production (in particular IgG2c), with a more prominent effect in newborn than adult mice (~400 as compared to ~150-fold increase, respectively) (Figure 2C). Interestingly, newborn mice immunized at DOL 7 and 14 (as indicated in Figure 2B) with (cGAMP + alum) still display the highest anti-rHA IgG and IgG2c titers at DOL 90 compared to saline and alum groups (Figure S8 in Supplementary Material). Enhancement of anti-rHA IgG and IgG2a titers induced by (cGAMP + alum) was also demonstrable in the Th2-skewed mouse strain BALB/c (Figure S9 in Supplementary Material).

In light of the robust adjuvanticity of the (cGAMP + alum) formulation, we quantified cGAMP adsorption to alum by RP-HPLC (Table 1). We observed a rapid initial adsorption of cGAMP onto alum (63% of total cGAMP) within 15 min from the incubation. The adsorption rate dropped quickly, with the overall adsorption reaching a plateau (75.33% of total cGAMP) after 24 h of incubation. No significant degradation products were observed over this time window.

**Table 1:** cGAMP adsorption onto alum as function of time as assessed by RP-HPLC.

cGAMP adsorbed onto alum—incubated at 37°C

Time point	Peak area (mAU)	% Adsorbed to alum
15 min	22.53	63.00
30 min	22.93	62.34
1 h	22.22	63.51
2 h	20.83	65.79
4 h	21.02	65.48
24 h	15.02	75.33
Alum control (no cGAMP)	0.53	ND
Saline	0.55	ND

*RP-HPLC, reverse-phase high performance liquid chromatography*.

Altogether, our *in vivo* results demonstrate that (cGAMP + alum) is an effective formulation to enhance antigen-specific antibody titers (especially of the IgG2a/c subclass) for early life immunization.

### (cGAMP + Alum) Fosters Th1 Polarization and GC Reaction

**Figure 3:**
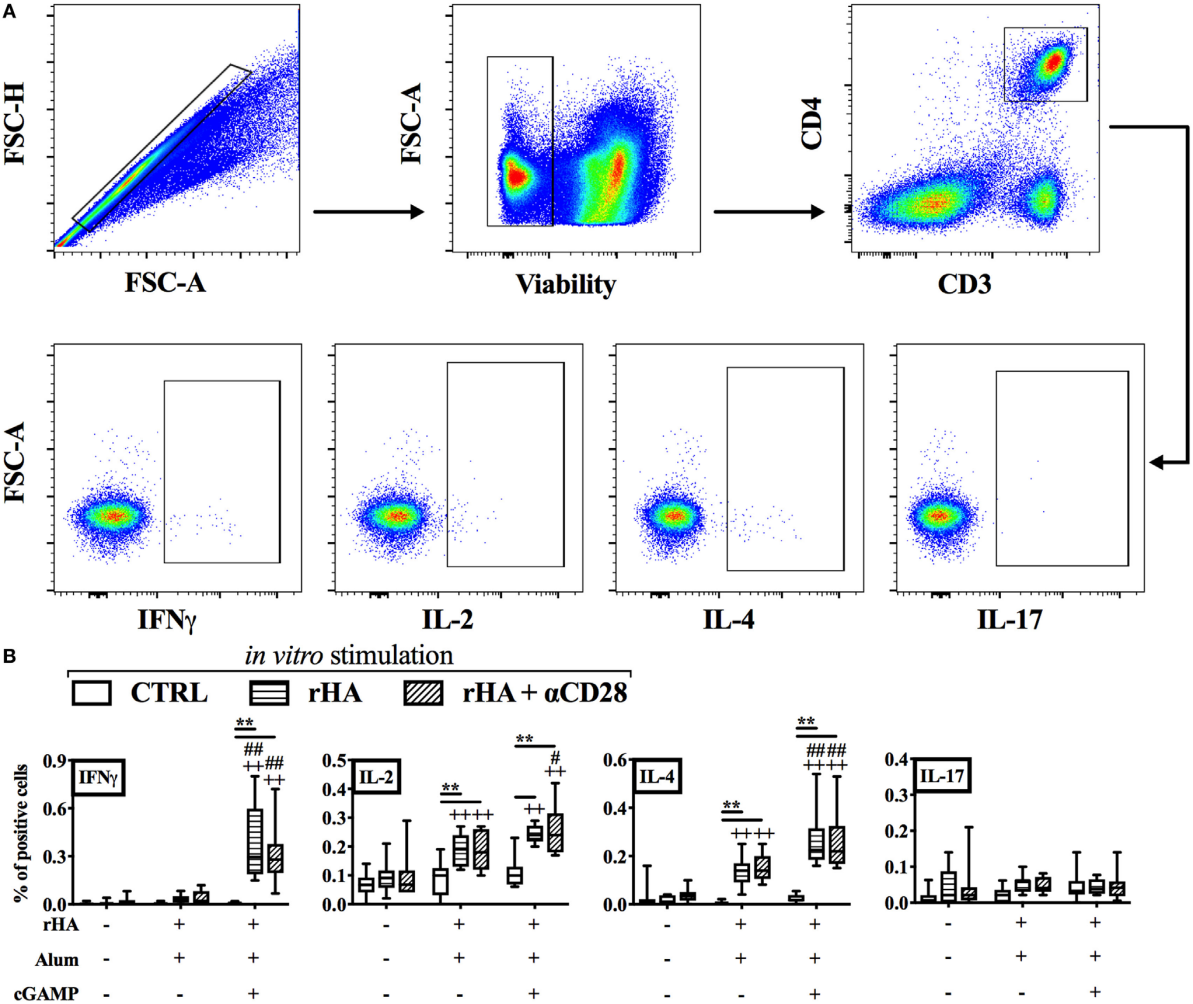
Immunization with (cGAMP + alum) induces Th1 polarization in early life. Newborn mice were immunized with alum or (cGAMP + alum) as indicated in Figure 2A. Ten days after boost [day of life (DOL) 24] splenocytes were harvested, re-stimulated for 18 h with recombinant hemagglutinin (rHA) in the presence or absence of the co-stimulus αCD28, and cytokine production by CD4^+^ T cells was assessed by intracellular flow cytometry. **(A)** Representative gating strategy. CD4^+^ T cells were defined as viable singlet CD3^+^ CD4^+^ cells. **(B)** Results are shown as the median, the 25th and 75th percentiles (boxes) and the 5th and 95th percentiles (whiskers) of 9–10 mice per group. **p < 0.01 of *in vitro* CTRL vs. rHA vs. rHA + αCD28, ^++^*p* < 0.01 of respective *in vitro* conditions compared to *in vivo* saline group, ^#^*p* < 0.05 and ^##^*p* < 0.01 of respective *in vitro* conditions compared to *in vivo* alum group, determined by two-way ANOVA with Tukey’s *post hoc* test.

**Figure 4:**
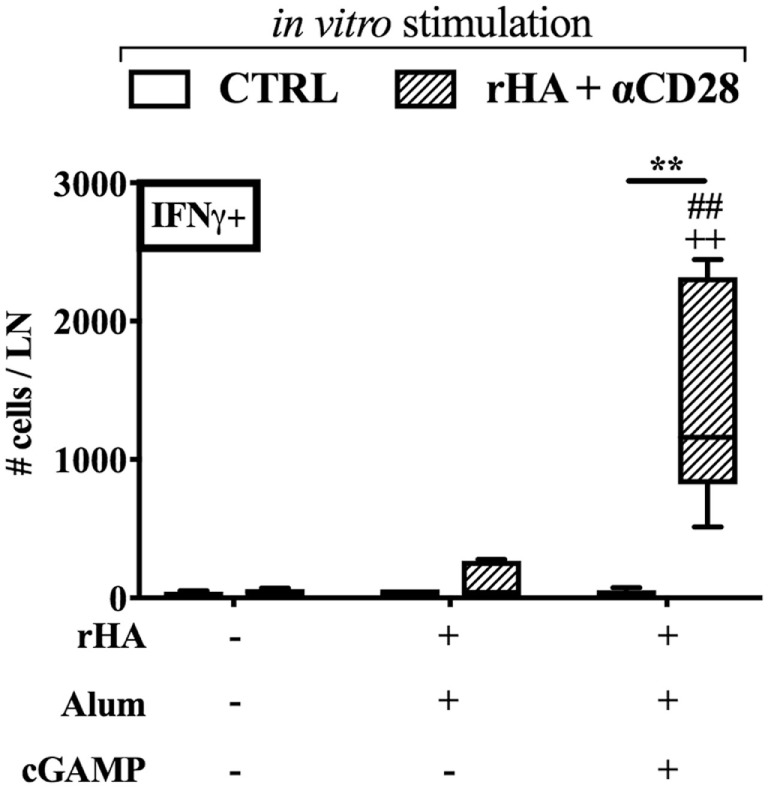
Immunization with (cGAMP + alum) induces IFNγ-producing cells in draining lymph nodes (dLNs) of newborn mice. Newborn mice were immunized with alum or (cGAMP + alum) as indicated in Figure 2A. 3 days after boost [day of life (DOL) 17] cells were isolated from dLNs, re-stimulated for 18 h with rHA + αCD28, and the number of IFNγ-producing cells per LN was assessed by ELISPOT. Results are shown as the median, the 25th and 75th percentiles (boxes) and the fifth and 95th percentiles (whiskers) of 4–5 mice per group. **p < 0.01 of *in vitro* CTRL vs. rHA + αCD28, ^++^*p* < 0.01 of respective *in vitro* conditions compared to *in vivo* saline group, ^##^*p* < 0.01 of respective *in vitro* conditions compared to *in vivo* alum group, determined by two-way ANOVA with Sidak’s *post hoc* test.

IgG2a/c isotype switching is driven by IFNγ *in vivo* (58), and reduced in early life, since newborns display reduced IFNγ production and Th1 polarization to many stimuli (6, 7). Therefore, we investigated whether (cGAMP + alum) was able to modulate the polarization and cytokine production of antigen-specific T cells. Accordingly, newborn mice were immunized as indicated in Figure 2A with alum or (cGAMP + alum). Ten days post-boost, splenocytes were harvested, re-stimulated with rHA in the presence or absence of the co-stimulus αCD28, and cytokine production by CD4^+^ T cells was measured by flow cytometry (Figure 3A). While IL-2- and IL-4-producing cells were observed in both groups, IFNγ^+^ CD4^+^ T (Th1) cells were only detected among splenocytes isolated from mice immunized with (cGAMP + alum) [median percentages of IFNγ^+^ CD4^+^ T cells upon rHA re-stimulation: 0.000 for saline, 0.031 for alum, and 0.295 for (cGAMP + alum) groups; upon rHA + αCD28 re-stimulation: 0.009 for saline, 0.021 for alum, and 0.280 for (cGAMP + alum) groups]. No IL-17 production was observed in any of the tested conditions (Figure 3B). To corroborate this evidence, upon *in vitro* re-stimulation with rHA + αCD28 we found by ELISPOT a higher number of IFNγ-producing cells in the dLNs of mice immunized with (cGAMP + alum) 3 days post-boost (Figure 4).

**Figure 5:**
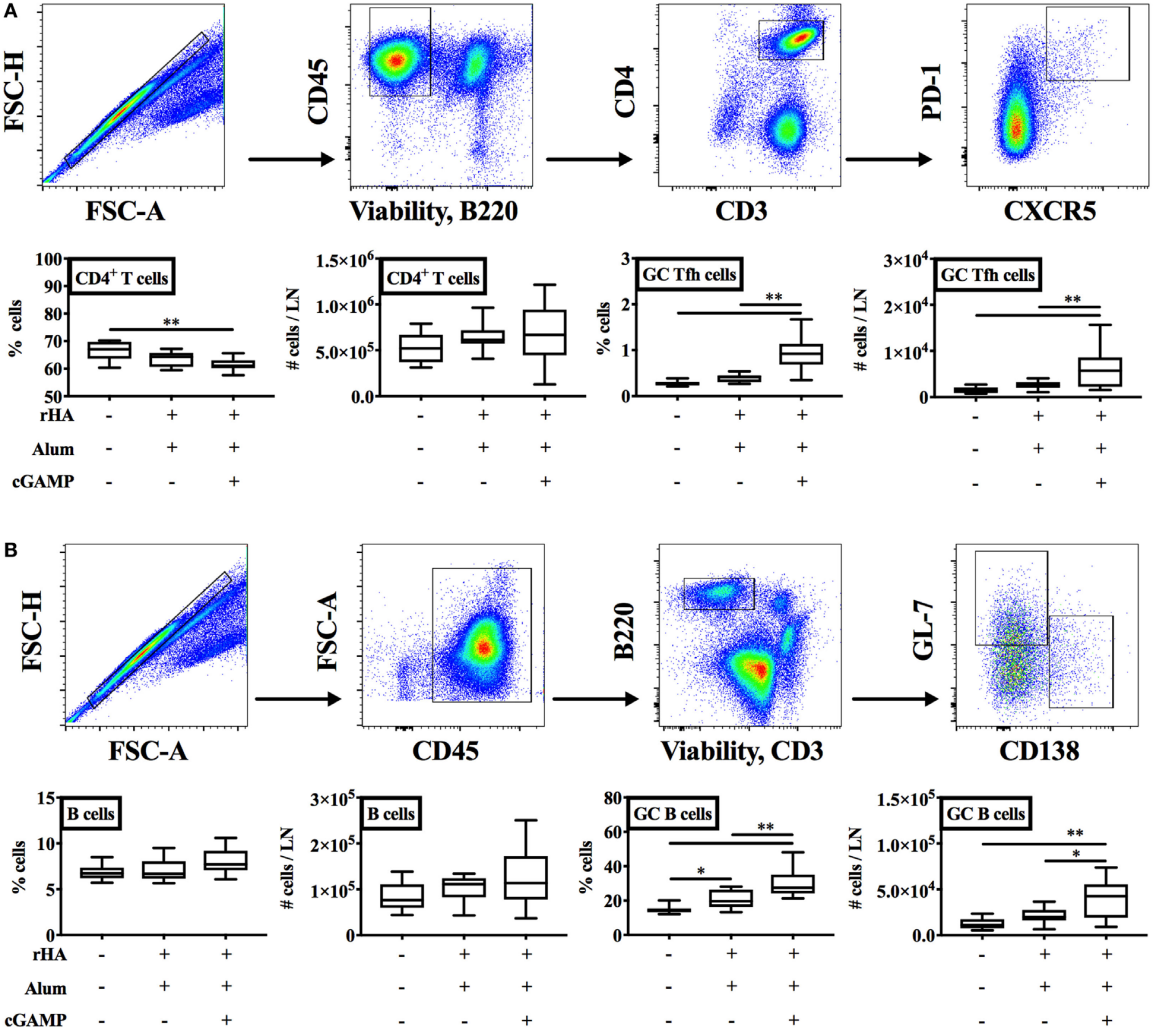
Immunization with (cGAMP + alum) fosters the germinal center (GC) reaction. Newborn mice were immunized with alum or (cGAMP + alum) as indicated in Figure 2A. Ten days after boost [day of life (DOL) 24] cells were isolated from draining lymph nodes and the percentages and absolute numbers of CD4^+^ T cells, B cells, GC Tfh, and B cells were assessed by flow cytometry. [**(A,B)** top panels] Representative gating strategies. CD4^+^ T cells were defined as viable singlet CD45^+^ B220^−^ CD3^+^ CD4^+^ cells. GC Tfh cells were defined as viable singlet CD45^+^ B220^−^ CD3^+^ CD4^+^ CXCR5^+^ PD-1^+^ cells. B cells were defined as viable singlet CD45^+^ B220^+^ CD3^−^ cells. GC B cells were defined as viable singlet CD45^+^ B220^+^ CD3^−^ GL-7^+^ CD138^−^ cells. **(B)** Results are shown as the median, the 25th and 75th percentiles (boxes) and the 5th and 95th percentiles (whiskers) of 9–10 mice per group. *p < 0.05, **p < 0.01 determined by one-way ANOVA with Holm–Sidak’s *post hoc* test.

T cell-dependent antibody generation is initiated in GCs and guided by Tfh cells (59, 60). Since GCs are major sites for isotype switching, we reasoned that immunization of newborn mice with (cGAMP + alum) might promote the GC reaction, thereby inducing IgG2a/c switching. To this aim, we assessed by flow cytometry the percentages and absolute numbers of GC Tfh and B cells (respectively, identified as viable singlet CD45^+^ B220^−^ CD3^+^ CD4^+^ CXCR5^+^ PD-1^+^ and CD45^+^ CD3^−^ B220^+^ GL-7^+^ CD138^−^ cells) in dLNs 10 days post-boost of newborn mice immunized with alum or (cGAMP + alum). Interestingly, we found a significant increase in the percentage [median: 0.275 for saline, 0.42 for alum, and 0.925 for (cGAMP + alum)] and absolute number [median: 1,360 for saline, 2,558 for alum, and 5,754 for (cGAMP + alum)] of GC Tfh cells and the percentage [median: 14.4 for saline, 19.7 for alum, and 27.35 for (cGAMP + alum)] and absolute number [median: 10,975 for saline, 19,878 for alum, and 42,524 for (cGAMP + alum)] of GC B cells only in the (cGAMP + alum) group (Figure 5A). Immunization with alum induced a small increase in the percentage (but not absolute number) of GC B cells, while only minor modifications of the percentages and absolute numbers of total CD4^+^ T cells and B cells were observed across different immunization groups (Figures 5A,B).

**Figure 6:**
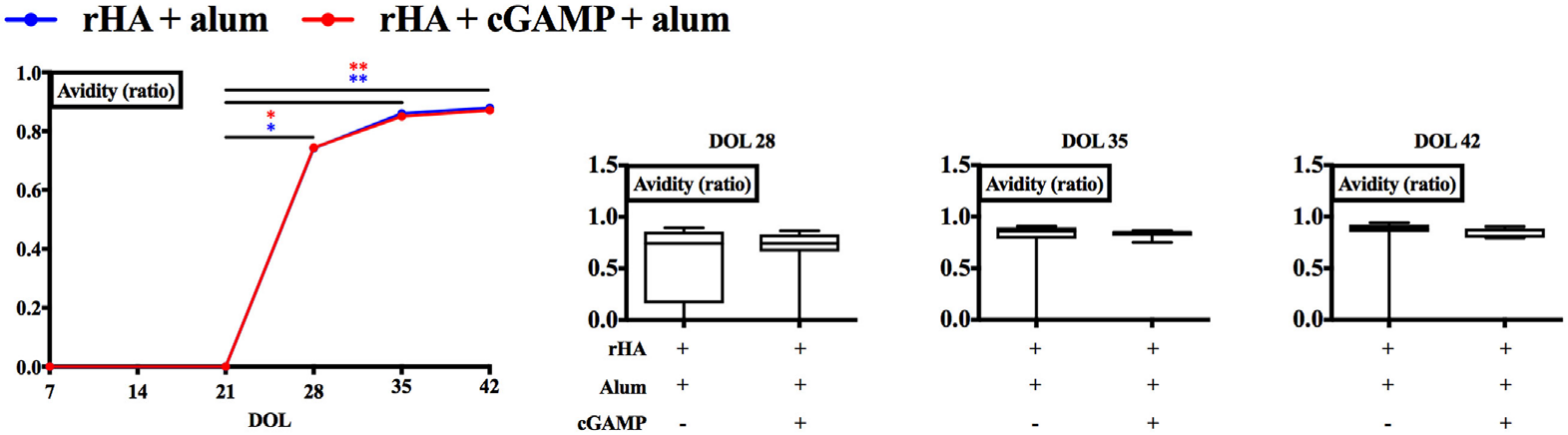
Immunization with (cGAMP + alum) does not modulate recombinant hemagglutinin (rHA)-specific IgG avidity. Newborn mice were immunized with rHA formulated with alum or (cGAMP + alum) and serum samples were collected as indicated in Figure 2. Avidity of rHA-specific IgG was measured by ELISA and expressed as the ratio between the LogEC50 values obtained with and without ammonium thiocyanate treatment (0.5 M). Results are shown as median (left panel) or as the median, the 25th and 75th percentiles (boxes) and the 5th and 95th percentiles (whiskers) (right panels) of 7–8 newborn mice per group. *p < 0.05, **p < 0.01 determined by two-way ANOVA with Sidak’s *post hoc* test (left panel) or Mann-Whitney test (right panels).

The GC is also the site where the processes of somatic hypermutation of antibody variable region genes and generation of high-affinity antibodies take place (60). To verify whether cGAMP modulates antibody affinity maturation, we measured rHA-specific IgG avidity of newborn mice immunized with alum or (cGAMP + alum) as indicated in Figure 2A. Although we observed a steep increase in antibody avidity 21 days post prime (DOL 28) which reached a plateau later on [28 (DOL 35) and 35 (DOL 42) days post-prime], no differences between the two groups were detected at any time point (Figure 6).

Overall, these results demonstrate that the addition of cGAMP to alum promoted the induction of IFNγ-producing T cells and appeared to foster the GC reaction, which might in turn drive IgG2a/c isotype switching in our early life immunization model.

### Single-Dose Immunization with (cGAMP + Alum) Induces rHA-Specific IgG2c Antibodies

**Figure 7:**
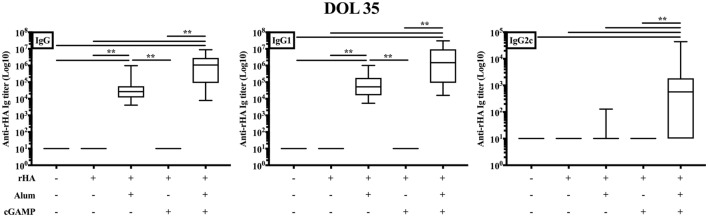
Single-dose immunization of newborn mice with (cGAMP + alum) significantly increases anti-recombinant hemagglutinin (rHA) IgG2c titers. Newborn mice were immunized i.m. with saline, rHA alone or formulated with alum, cGAMP or (cGAMP + alum) and antibody titers for rHA-specific IgG, IgG1, and IgG2c were determined by ELISA in serum samples collected 28 days after boost [day of life (DOL) 35]. Results are shown as the median, the 25th and 75th percentiles (boxes), and the 5th and 95th percentiles (whiskers) of 9–13 mice per group. **p < 0.01 determined by Kruskal–Wallis with Dunn’s *post hoc* test.

The results obtained so far supported the efficacy of (cGAMP + alum) as an adjuvantation system in a prime/boost model of neonatal murine immunization. Of note, a single-dose immunization strategy capable of enhancing antigen-specific antibody titers would be highly desirable early in life. To this end, we immunized newborn mice with rHA formulated with alum, cGAMP, or (cGAMP + alum). Distinct from its effects in prime/boost immunization, cGAMP without alum did not induce detectable anti-rHA IgG, IgG1, and IgG2c titers. Alum and (cGAMP + alum) significantly increased anti-rHA IgG and IgG1 titers [median anti-rHA IgG and IgG1 titers: respectively, 26.74 × 10^3^ and 52.08 × 10^3^ for alum; respectively, 1.07 × 10^6^ and 1.48 × 10^6^ for (cGAMP + alum)]. Interestingly, only (cGAMP + alum) induced detectable levels of anti-rHA IgG2c (median: 571.9), albeit at lower levels compared to prime/boost immunization (Figure 7). Altogether, these results demonstrate that (cGAMP + alum) is an effective adjuvantation system also for single dose early life immunization.

## Discussion

Over the past decades, many PRRs and their agonists have been identified, and the molecular definition of their mechanisms of action and immunostimulatory properties has paved the way for new classes of adjuvants (26, 61). For example, the TLR4 agonist monophosphoryl lipid A is employed in different FDA-approved vaccine formulations. Despite this wealth of knowledge, the portfolio of adjuvants approved or in clinical development for the newborn and the young infant is much narrower, in part due to our limited knowledge of the immune system early in life (6, 7, 62). Notwithstanding these limitations, *in vitro* and pre-clinical *in vivo* studies have shown that targeting some PRRs, in particular TLR7/8 (8–14), potently activates newborn immune cells and markedly enhances vaccine efficacy early in life. Here, by combining an *in vitro* analysis of newborn BMDC activation in response to PRR agonists and *in vivo* immunization models, we identify the STING agonist cGAMP as adjuvant candidate for early life immunization. In particular, we demonstrate that immunization of newborn mice with cGAMP formulated with alum appears to foster the GC reaction as well as features of IFNγ-driven type 1 immunity, namely switching toward IgG2a/c subclass and Th1 polarization.

Although there is no comprehensive consensus on whether and how *in vitro* models can predict the *in vivo* effect of candidate adjuvants, the use of DCs has some advantages for assessing their activity *in vitro* (6, 27, 63, 64). First, DCs are the most prominent subset of antigen-presenting cells. Second, they express many PRRs. Third, DCs can be employed to recapitulate age-specific differences. Although isolating primary DCs from spleen and lymph nodes of neonatal mice would be ideal, this approach is cumbersome if not impossible due to low cell yield (65, 66). Therefore, we developed and characterized a neonatal BMDC model, and found phenotypic and functional differences between neonatal and adult BMDCs. Most importantly, by comparing the activation profiles of neonatal and adult BMDCs we identify that the STING agonist cGAMP induces their maturation (e.g., upregulation of CD40, CD80, and CD86). Although we proceeded in assessing the *in vivo* adjuvant activity of cGAMP, we cannot exclude that other PRR ligands that did not activate newborn BMDCs *in vitro* might act as adjuvants *in vivo*. Therefore, further studies, especially of combination formulations, are required to define the predictive value of the *in vitro* newborn BMDC system.

Cyclic dinucleotides including cGAMP have been tested as candidate adjuvants in experimental models of parenteral or mucosal adult immunization (32–49). In the present work, mice were immunized by the intramuscular route as it is commonly employed for pediatric vaccines: a new formulation specific for intramuscular injection may fit easily with other vaccines in the pediatric vaccination schedule, while intranasal immunization against influenza virus, for example, is currently not recommended by the CDC (67). We found that free cGAMP, simply injected together with the model antigen, is much less effective in newborn than in adult mice at increasing antigen-specific antibody titers. Remarkably, cGAMP formulated with alum induces relatively high titers of antigen-specific IgG2a/c compared to alum or cGAMP alone, especially in newborn mice immunized with prime/boost or single dose schedules. The explanation for this might be that about 60% of cGAMP adsorbs onto alum *in vitro*, which also suggests that there is still the possibility of further optimizing this formulation and increasing the percentage of adsorbed cGAMP by modification of the adsorption pH, buffer, and alum to cGAMP ratio. Interestingly, it has already been reported that CDNs tend to diffuse in the bloodstream after injection, while their nanoparticle formulations deliver CDNs to the dLNs (40). It is tempting to speculate that the same phenomenon might explain the differences in the efficacy between cGAMP and (cGAMP + alum). In addition, it will be interesting to compare the effect of optimized (cGAMP + alum) and nanoparticle-based cGAMP formulations in our early life immunization model.

Newborns and young infants have a distinct immunity with an impairment of IFNγ-driven type 1 immunity, which in turn leads to reduced vaccine efficacy and higher risk of infections (6, 7). By using (cGAMP + alum) as adjuvantation strategy for early life immunization, we were able to induce cardinal features of type 1 immunity: (1) IFNγ production by antigen-specific CD4^+^ T cells and (2) relatively high titers of antigen-specific IgG2a/c. As IFNγ promotes isotype switching toward IgG2a/c *in vivo* (58), these two events are likely linked. The importance of inducing this antibody subclass relies in its higher affinity toward Fcγ receptors expressed on myeloid cells, which endows this subclass with greater effector functions (e.g., induction of phagocytosis, complement fixation) that may be important for protecting from infections (56, 57). Our results also suggest that (cGAMP + alum) increases the magnitude of the GC reaction, known to be impaired in early life (24, 25), by inducing higher percentages and absolute numbers of GC Tfh and B cells in dLNs. Although we cannot exclude that the GC reaction induced by alum follows a different kinetics, these results might represent the cellular correlate of the isotype switching and early IgG2a/c production observed in the (cGAMP + alum) group. Altogether, our data point to a relevant effect of the (cGAMP + alum) formulation on the humoral and cellular immune responses elicited upon immunization early in life.

Overall, our study features several strengths, including (a) the first immunophenotypic characterization of murine neonatal BMDCs, (b) an unbiased screening of PRR agonists for activity toward neonatal BMDCs, and (c) identification of a novel adjuvantation system active *in vitro* and *in vivo* with evidence supporting potential utility in enabling single-dose immunization at birth. Our study also has limitations, including (a) the neonatal BMDC model studied represents a mix of cells generated by treatment with cytokines *in vitro* such that they may not fully reflect *in vivo* biology, (b) the potential effects of (cGAMP + alum) on GCs are intriguing but until such time as they are verified by microscopy are inferential, (c) although our studies demonstrated robust increases in antibody titers and features of type 1 immunity elicited by immunization with (cGAMP + alum), future functional studies (e.g., pathogen challenge) are required to assess the efficacy of this adjuvantation system, and (d) due to species specificity, results in mice may not accurately reflect those in humans.

In conclusion, we demonstrate that cGAMP is a promising and robust adjuvant candidate for early life immunization. We also show that cGAMP formulated with alum potently enhances humoral and cellular aspects of type 1 immunity in early life. Since we employed the rHA influenza vaccine throughout our work, our results may be applicable to influenza immunization. Use of (cGAMP + alum) may also represent a general strategy to elicit type 1 immunity toward protein antigens for early life immunization.

## Ethics Statement

All experiments involving animals were approved by the Animal Care and Use Committee of Boston Children’s Hospital and Harvard Medical School (protocol numbers 15-11-3011 and 16-02-3130).

## Author Contributions

FB, CP, DD, and OL designed the study. CP, DD, and FB conducted the *in vitro* experiments. FB, CP, and JL conducted the *in vivo* experiments. LW and DB conducted the adsorbance experiments. FB and CP wrote the manuscript. DD and OL provided overall mentorship and assisted in writing the manuscript. FB, CP, JL, LW, PS, DO, JR, SB, LP, FM, DB, DD, and OL contributed to helpful discussions, review, and approval of the final manuscript. All the authors have given final approval for the version submitted for publication.

## Conflict of Interest Statement

The authors declare that the research was conducted in the absence of any commercial or financial relationships that could be construed as a potential conflict of interest. The reviewer JW declared a shared affiliation, though no other collaboration, with several of the authors (FB, CP, JL, PS, DD, and OL) to the handling editor.
