# Supplementary material for: Identification and Characterization of Stimulator of Interferon Genes As a Robust Adjuvant Target for Early Life Immunization
Source: Front Immunol. 2017 Dec 12;8:1772. doi: 10.3389/fimmu.2017.01772 (PMC5732947; doi:10.3389/fimmu.2017.01772)
Supplement: Supplementary file 1 [file Data_Sheet_1.PDF]

Table S1. List of fluorochromes and antibodies used in the study for flow cytometry stainings

Target	Clone	Fluorochrome	Dilution	Company
**CD11c**	HL3	BV421	1:200	BD Biosciences
**CD11c**	N418	Alexa-700	1:400	Biolegend
**MHCII**	M5/114	APC	1:200	Biolegend
**CD40**	3/23	PE-Dazzle 594	1:50	Biolegend
**CD80**	16-10A1	Alexa Fluor 488	1:50	Biolegend
**CD86**	GL-1	PE	1:150	BD Biosciences
**CD11b**	M1/70	Alexa Fluor 488	1:400	Biolegend
**CD115**	AFS98	Alexa Fluor 488	1:100	Biolegend
**CD117**	2B8	BV421	1:40	Biolegend
**CD135**	A2F10	PE	1:40	Biolegend
**CD64**	X54-5/7.1	PerCP-Cy5.5	1:200	Biolegend
**F4/80**	BM8	APC	1:200	eBioscience
**CD45**	30-F11	PerCP-Cy5.5	1:80	Biolegend
**IFNγ**	XMG1.2	APC	1:40	Biolegend
**IL-2**	JES6-5H4	BV421	1:40	Biolegend
**IL-4**	11B11	PE	1:40	Biolegend
**IL-17A**	TCH18-H10	PE-CF594	1:40	BD Biosciences
**CD3**	17A2	FITC	1:40	BD Biosciences
**CD3**	17A2	APC-Fire750	1:40	Biolegend
**CD4**	GK1.5	PerCP-Cy5.5	1:40	Biolegend
**CXCR5**	L138D7	BV421	1:40	Biolegend
**PD-1**	29F.1A12	PE	1:40	Biolegend
**B220**	RA3-6B2	Alexa Fluor 488	1:40	Biolegend
**B220**	RA3-6B2	APC-eFluor780	1:40	eBioscience
**CD138**	281-2	BV421	1:40	Biolegend
**GL-7**	GL7	PE	1:80	Biolegend
**Viability**		eFluor 780	1:1000	eBioscience
**Mouse BD Fc Block**	2.4G2		1:80	BD Biosciences

Table S2: List of PRRs agonists used to stimulate BMDCs from newborn and adult mice

Receptor	Agonist Name	Concentration Range
Toll Like Receptors (TLR) agonists		
TLR1	PAM3CSK4	1, 10, **100** ng/ml
TLR2	PAM2CSK4	1, 10, **100** ng/ml
TLR3	Poly (I:C) HMW	1, 10, **100** ng/ml
TLR4	Synthetic monophosphoryl Lipid A (MPLA)	1, 10, **100,** 1000 ng/ml
TLR5	Flagellin S.t. ultrapure	1, 10, **100** ng/ml
TLR2/6	FSL-1	1, 10, **100** ng/ml
TLR7	CL264	0.01, 0.1, **1**, 10 μM
TLR7/8	R848	0.01, **0.1**, 1, 10 μM
TLR8/7	CL075	0.01, **0.1**, 1, **10** μM
TLR8	TL8-506	0.01, 0.1, 1, **10** μM
TLR9	CpG class C - ODN 2395	0.01, 0.1, **1**, 10 μM
NOD-like receptor (NLR) agonist		
NOD1	C12-iE-DAP	1, 10, **100** ng/ml
NOD2	L18-MDP	1, 10, **100** ng/ml
C-type Lectin Receptor agonists		
Dectin-1	Curdlan (β-glucan)	0.1, 1, **10** μg/ml
Dectin-2	Furfurman	**0.1**, 1, 10 μg/ml
MINCLE	TDB	0.1, **1**, 10 μg/ml
Retinoic acid-inducible gene (RIG)-I-like receptor (RLRs) agonists		
RIG-I	5'ppp-dsRNA	10, 100, **1000** ng/ml
RIG-I	Poly (dA:dT)	10, 100, **1000** ng/ml
Inflammasome inducers		
NLRP3	Alum phosphate (Adju-Phos)	0.5, 5, **50** μg/mL
NLRP3	Alum hydroxide (Alhydrogel)	0.5, 5, **50** μg/mL
STING agonists		
STING	2'3'-cGAMP	1, 10, **100** μg/ml
STING/NLRP3	Chitosan	1, **10**, 100 μg/ml

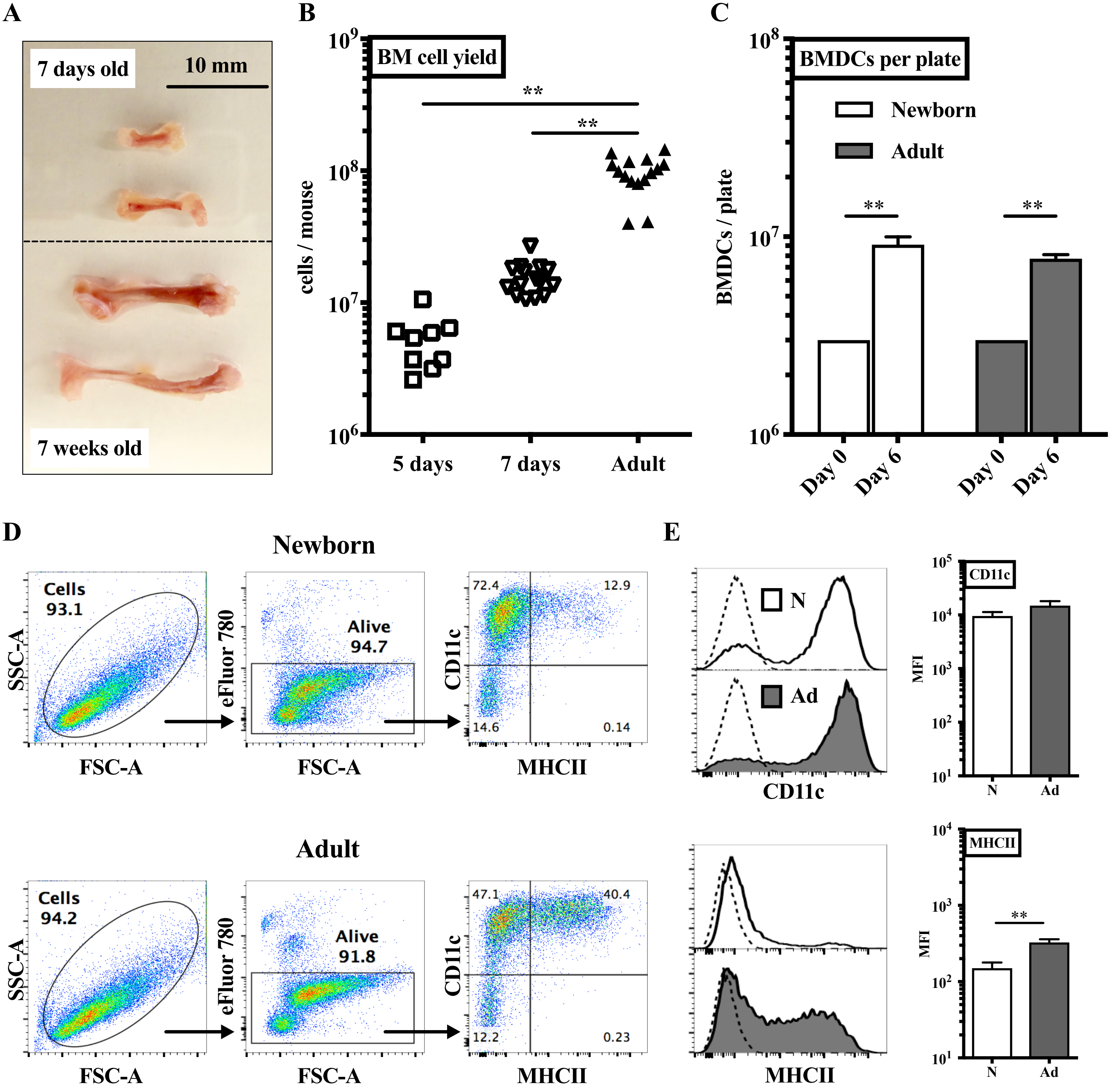
A
7 days old
10 mm
7 weeks old
B
BM cell yield
cells / mouse
5 days
7 days
Adult
C
BMDCs per plate
Newborn
Adult
BMDCs / plate
Day 0
Day 6
Day 0
Day 6
D
Newborn
Cells
93.1
SSC-A
FSC-A
eFluor 780
Alive
94.7
CD11c
MHCII
72.4
12.9
14.6
0.14
Adult
Cells
94.2
Alive
91.8
47.1
40.4
12.2
0.23
E
N
Ad
CD11c
MHCII
MFI
N
Ad

Figure S1

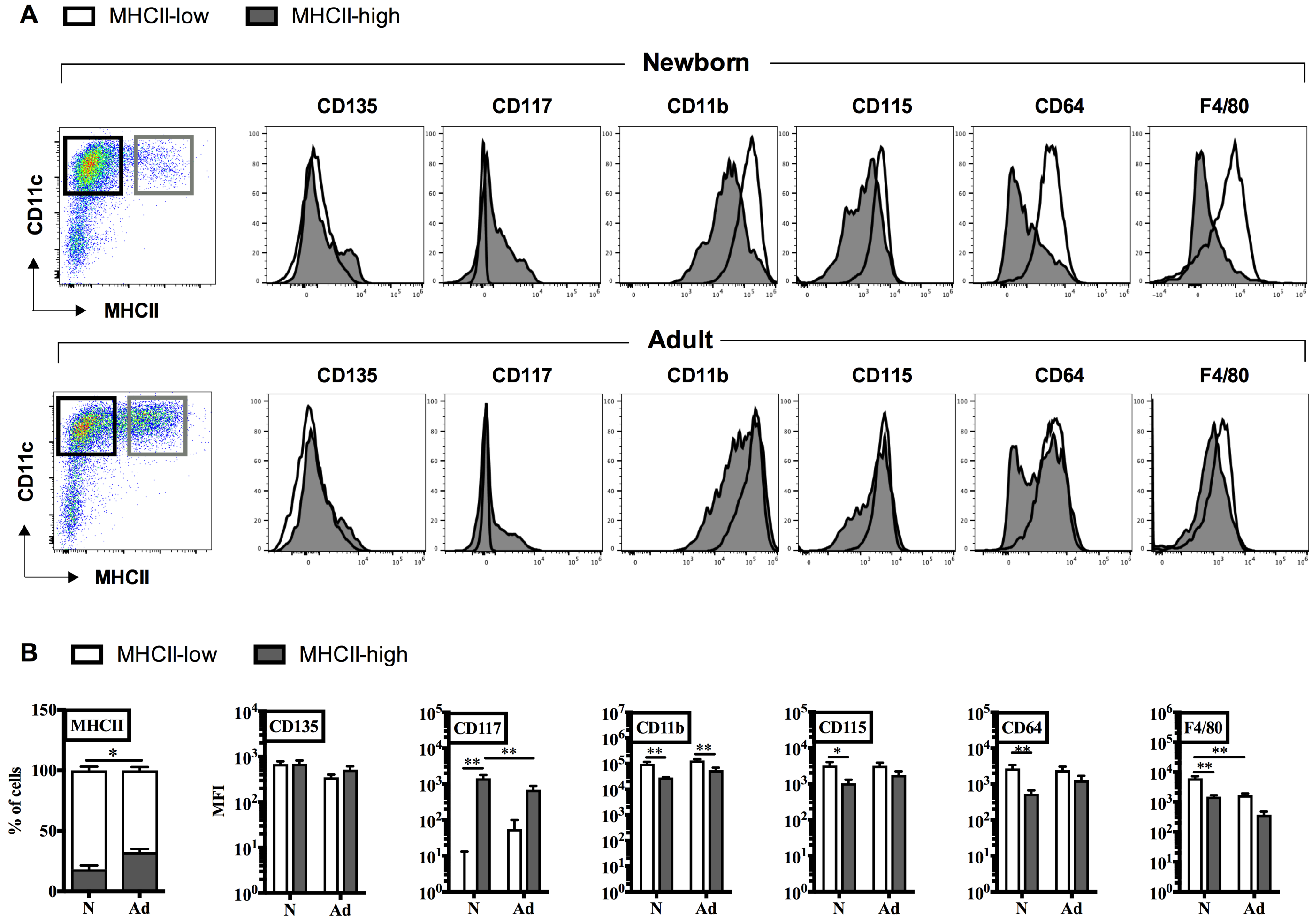

A
MHCII-low
MHCII-high
Newborn
CD135
CD117
CD11b
CD115
CD64
F4/80
CD11c
MHCII
Adult
CD135
CD117
CD11b
CD115
CD64
F4/80
CD11c
MHCII
B
MHCII-low
MHCII-high
MHCII
% of cells
CD135
MFI
CD117
CD11b
CD115
CD64
F4/80
N
Ad

Figure S2

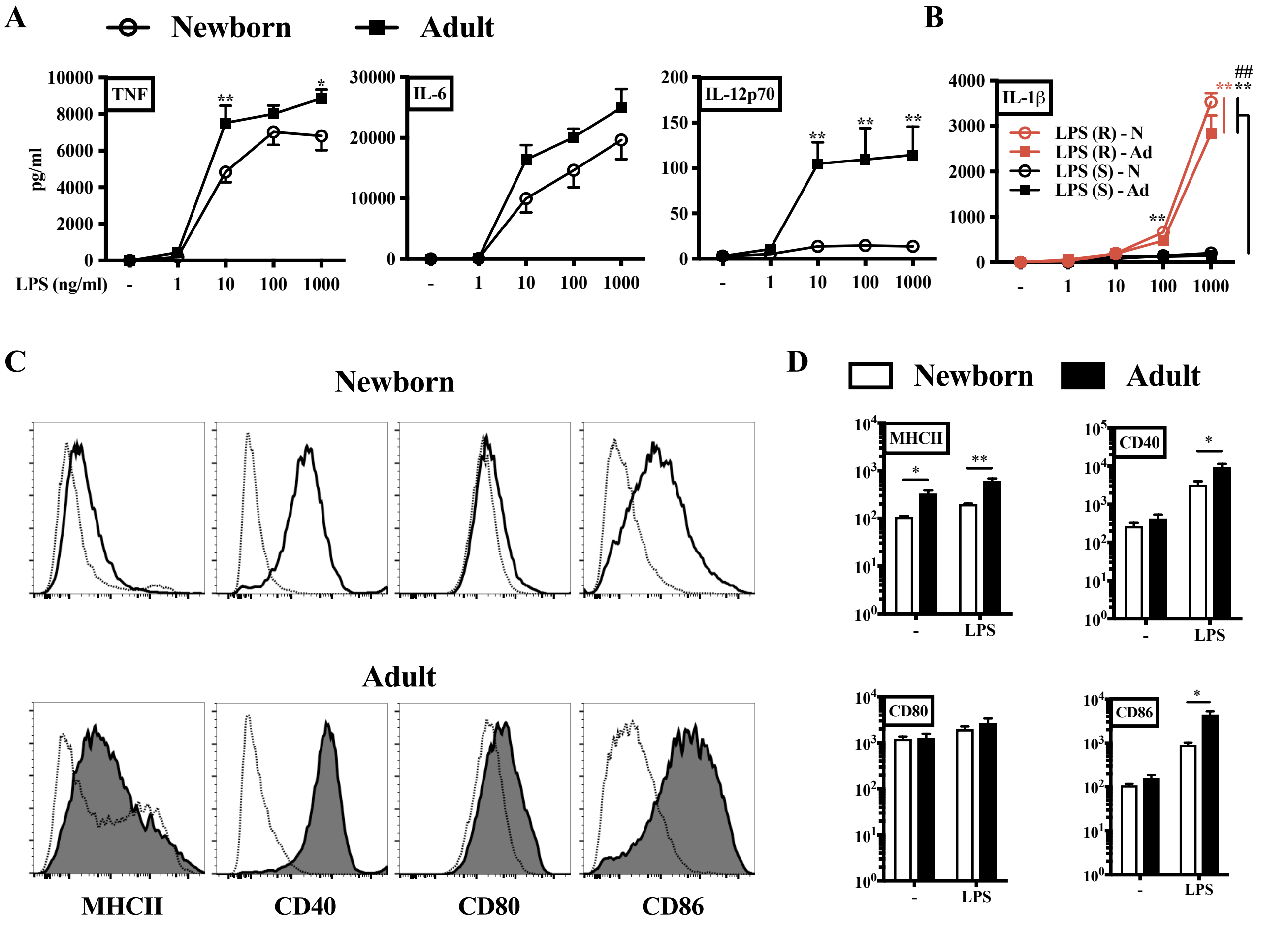
A
Newborn
Adult
TNF
IL-6
IL-12p70
pg/ml
LPS (ng/ml)
- 1 10 100 1000
B
IL-1β
LPS (R) - N
LPS (R) - Ad
LPS (S) - N
LPS (S) - Ad
##
C
Newborn
Adult
MHCII
CD40
CD80
CD86
D
Newborn
Adult
MHCII
CD40
CD80
CD86
LPS

Figure S3

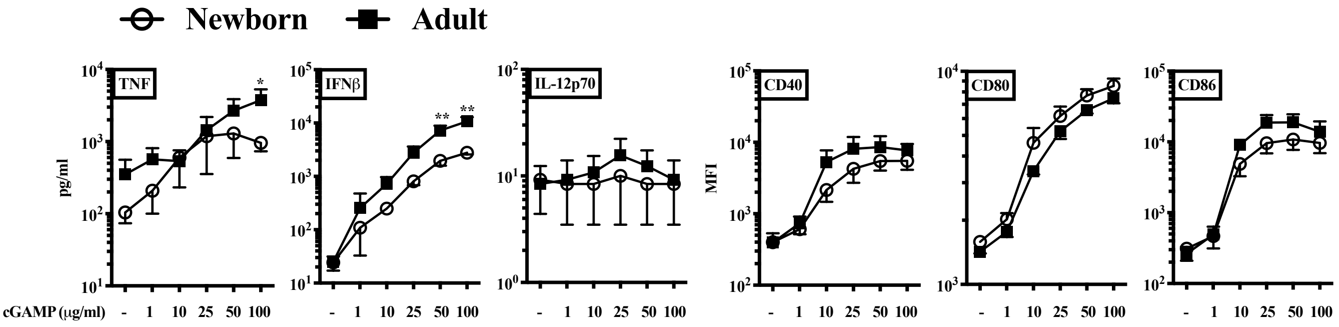
Newborn
Adult
TNF
IFNβ
IL-12p70
CD40
CD80
CD86
pg/ml
MFI
cGAMP (μg/ml)
- 1 10 25 50 100
*
**
**

Figure S4

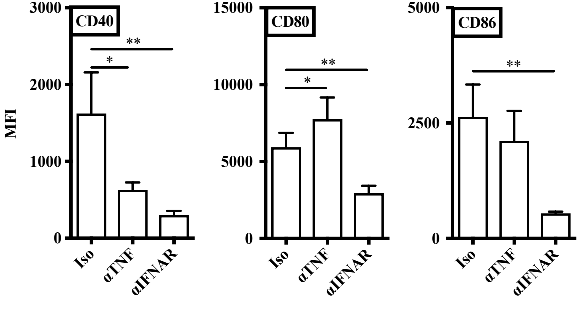

CD40
CD80
CD86
MFI
3000
2000
1000
0
15000
10000
5000
0
5000
2500
0
*
**
*
**
**
Iso
αTNF
αIFNAR
Iso
αTNF
αIFNAR
Iso
αTNF
αIFNAR

Figure S5

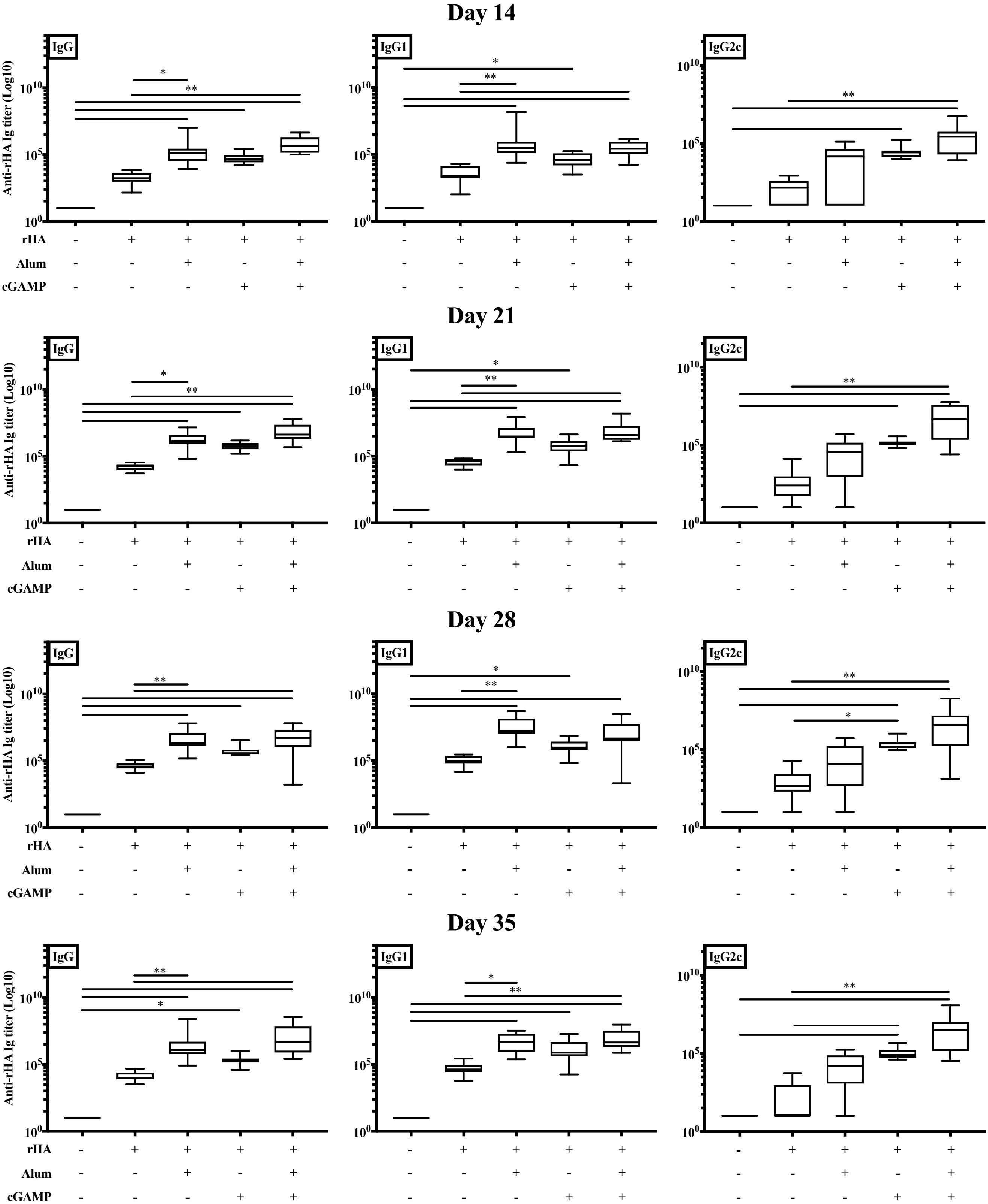
Day 14
Day 21
Day 28
Day 35
IgG
IgG1
IgG2c
Anti-rHA Ig titer (Log10)
rHA
Alum
cGAMP
*
**

Figure S6

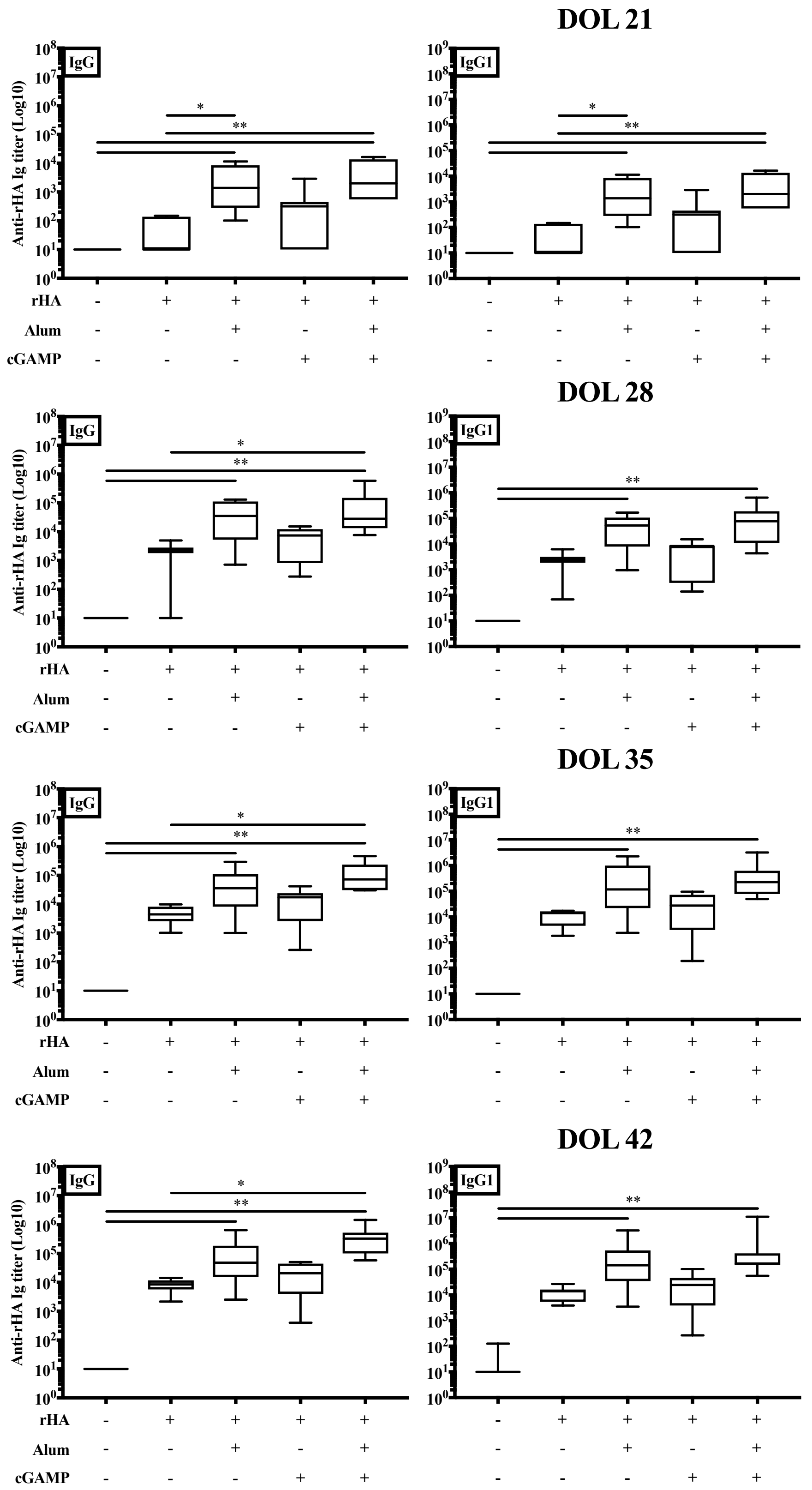
DOL 21
DOL 28
DOL 35
DOL 42
IgG
IgG1
IgG2c
Anti-rHA Ig titer (Log10)
rHA
Alum
cGAMP

Figure S7

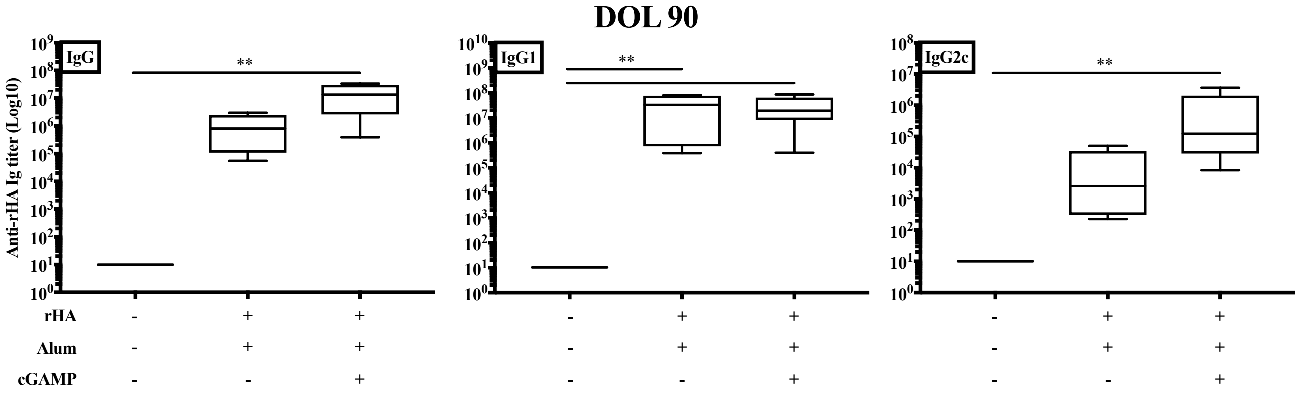

Figure S8

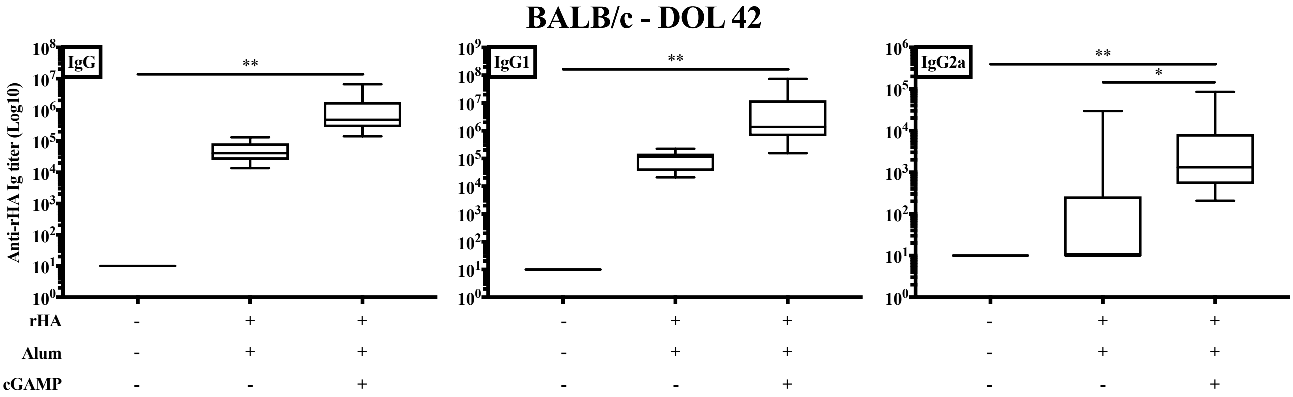
BALB/c - DOL 42
Anti-rHA Ig titer (Log10)
IgG
IgG1
IgG2a
**
*
rHA
Alum
cGAMP

Figure S9

Figure S1. Characterization of neonatal GM-CSF-differentiated bone marrow-derived dendritic cells. **(A)** Representative picture of tibia and femur from 7 day old (top) and 8 week old (bottom) mice. **(B)** Bone marrow cell yield at different ages from 4 bones per mouse. **(C)** *In vitro* expansion of BM precursors into BMDCs after 6 days of culture for newborn (white) and adult (gray) mice. **(D)** Representative gating strategy used to identify neonatal (top) and adult (bottom) BMDCs. **(E)** Median fluorescence intensity (MFI) of CD11c and MHCII expression by newborn (N, white) and adult (Ad, gray) BMDCs. Dotted line, unstained controls. Results are shown as scatter dot plot **(B)** or as mean + SEM **(C, E)** of 9-15 mice **(B)**, 17-24 **(C)** or 6 **(E)** independent experiment. * $p < 0.01$ determined by one-way ANOVA with Tukey's post hoc test **(B)** or unpaired t test **(C, E)**.

Figure S2. Neonatal and adult BMDCs express distinct surface phenotypes. **(A)** Representative gating strategy and surface marker expression of MHCII-high (open histograms) and MHC-low (grey histograms) BMDCs generated from newborn (top) and adult (bottom) mice. **(B)** Percentage of MHCII-high and -low BMDCs generated from adult and newborn mice (left panel) and MFI of myeloid marker expression by neonatal and adult MHCII-high (gray bars) and -low (white bars) BMDCs. Results are expressed as mean + SEM of 4 independent experiments. * $p < 0.05$, ** $p < 0.01$ determined by two-way ANOVA with Sidak post hoc test.

Figure S3. Neonatal and adult BMDCs exhibit distinct cytokine and phenotypic profiles upon LPS stimulation. Neonatal and adult BMDCs were stimulated with smooth **(A-D)** or rough **(B**, red lines and symbols**)** LPS for 20-24 hours. Cytokine production **(A, B)** and MFI of surface marker expression (**C, D)** were respectively assessed by ELISA and flow cytometry. **(C)** Representative histograms of surface marker expression. Results are expressed as mean + SEM of 6 **(A, B)** or 4 **(D)** independent experiments. * $p < 0.05$, ** $p < 0.01$ determined by two-way ANOVA with Sidak post hoc test.

Figure S4. Neonatal and adult BMDC cytokine production and surface maturation marker expression upon stimulation with cGAMP. Neonatal (open circles) and adult (black boxes) BMDCs were stimulated for 20-24 hours with increasing concentrations of cGAMP. Cytokine production and surface expression of maturation markers were respectively assessed by ELISA and flow cytometry. Results are expressed as mean + SEM of 4-5 (cytokine production) or 3 (surface marker expression) independent experiments. * $p < 0.05$, ** $p < 0.01$ determined by repeated measures two-way ANOVA with Sidak post hoc test.

Figure S5. cGAMP induces neonatal BMDC maturation in a type I interferon-dependent manner. Neonatal BMDCs were stimulated for 20-24 hours with cGAMP (25 µg/ml) in the presence of blocking anti-TNF and anti-IFNAR antibodies or an isotype control. Surface expression of maturation markers was assessed by flow cytometry. Results are expressed as mean + SEM of 3 independent experiments. * $p < 0.05$, ** $p < 0.$ determined by repeated measures one-way ANOVA with Dunnett's post hoc test.

Figure S6. Antibody titers in adult mice immunized with rHA formulated with cGAMP and alum. Adult mice were immunized and antibody titers were determined as indicated in Figure 2. Results are shown as the median, the 25th and 75th percentiles (boxes) and the 5th and 95th percentiles (whiskers) of 9-10 mice per group. * $p < 0.05$, ** $p < 0.01$ determined by Kruskall-Wallis with Dunn's post hoc test.

Figure S7. Antibody titers in newborn mice immunized with rHA formulated with cGAMP and alum. Newborn mice were immunized and antibody titers were determined as indicated in Figure 2. Results are shown as the median, the 25th and 75th percentiles (boxes) and the 5th and

95th percentiles (whiskers) of 7-8 mice per group. * $p < 0.05$, ** $p < 0.01$ determined by Kruskall-Wallis with Dunn's post hoc test.

Figure S8. Immunization with cGAMP + alum induces long-term persistence of rHA-specific IgG2c. Newborn mice were immunized and antibody titers were determined at DOL 90 as indicated in Figure 2. Results are shown as the median, the 25th and 75th percentiles (boxes) and the 5th and 95th percentiles (whiskers) of 5 mice per group. ** $p < 0.01$ determined by Kruskall-Wallis with Dunn's post hoc test.

Figure S9. Immunization of newborn BALB/c mice with rHA formulated with cGAMP and alum enhances rHA-specific antibody titers. Newborn BALB/c mice were immunized and antibody titers were determined at DOL 42 as indicated in Figure 2. Results are shown as the median, the 25th and 75th percentiles (boxes) and the 5th and 95th percentiles (whiskers) of 7-8 mice per group. ** $p < 0.01$ determined by Kruskall-Wallis with Dunn's post hoc test.
